# Bioactivity Assessment of Indian Origin—Mangrove Actinobacteria against *Candida albicans*

**DOI:** 10.3390/md16020060

**Published:** 2018-02-12

**Authors:** J. G. S. Pavan Kumar, Ajitha Gomathi, Vitor Vasconcelos, K. M. Gothandam

**Affiliations:** 1 Department of Biotechnology, School of Biosciences and Technology, VIT University, Vellore 632014, India; pavankumarup6@gmail.com (J.G.S.P.K.); aji.mku@gmail.com (A.G.); gothandam@gmail.com (K.M.G.); 2 CIIMAR/CIMAR—Interdisciplinary Centre of Marine and Environmental Research, University of Porto, Terminal de Cruzeiros do Porto de Leixões, 4450-208 Matosinhos, Portugal; 3 Department of Biology, Faculty of Sciences, University of Porto, 4069-007 Porto, Portugal

**Keywords:** actinobacteria, antifungal, polyketide synthase, non ribosomal peptide synthase, *Candida albicans*

## Abstract

Actinobacteria is found to have a potent metabolic activity against pathogens. The present study reveals the assessment of potent antifungal secondary metabolites from actinobacteria isolated from Indian marine mangrove sediments. The samples were collected from the coastal regions of Muthupet, Andaman and the Nicobar Islands. Identification was carried out using 16S rRNA analysis and biosynthetic genes (Polyketide synthase type I/II and Non-ribosomal peptide synthase) were screened. Actinobacteria were assayed for their antifungal activity against 16 clinical *Candida albicans* and the compound analysis was performed using gas chromatography-mass spectrometry GC-MS. The 31 actinobacterial strains were isolated and 16S rRNA gene sequencing revealed that this ecosystem is rich on actinobacteria, with *Streptomyces* as the predominant genus. The PCR based screening of biosynthetic genes revealed the presence of PKS-I in six strains, PKS-II in four strains and NRPS in 11 strains. The isolated actinobacteria VITGAP240 and VITGAP241 (two isolates) were found to have a potential antifungal activity against all the tested *C. albicans*. GC-MS results revealed that the actinobacterial compounds were belonging to heterocyclic, polyketides and peptides. Overall, the strains possess a wide spectrum of antifungal properties which affords the production of significant bioactive metabolites as potential antibiotics.

## 1. Introduction

Bacterial cell factories and their wide applications in search of added value products such as molecular therapeutics is booming currently; they have a promising future as the safest and most efficient medicinal product discovery [1]. Flora and fauna as well as microorganisms are good sources to obtain a wide variety of natural medicinal products [2,3]. Actinobacteria are very significant in this regard [4], taken into account their intrinsic pharmacological importance [5]. The secondary metabolites produced by these gram positive bacteria, have a wide spectrum of activities such as antimicrobial, antiviral, immune-modulatory and as anticancer agents [6]. Peptides and polyketides are noticeable and they are a potential group of natural products, mainly synthesized by two major types of enzymes: non-ribosomal peptide synthases (NRPS) and polyketide synthases (PKS) [7]. Microbes harbouring these biosynthetic gene clusters are probable source of novel natural products. Polyketides have extensive applications as antibiotics in the pharmaceutical industry [8]. With several classes of PKS genes known, type I PKS encompasses multidomain enzymes and type II PKS is comprised of many enzymes [9]. NRPS are modular enzymes with multiple domains, namely acetylation, condensation, and thioesterase [10]. Analysing the biosynthetic genes depicts the potential of the microbe to produce a particular type of natural product [11]. Moreover, gene guided metabolite studies also help in the avoidance of the redundant discovery of known compounds [12]. These genes have served to be a great source of interesting natural products with pharmacological relevance [1].

Apart from traditional techniques, such as identification or screening of pharmacologically important microbes isolated from various ecosystems, the 16S rRNA phylogenetic analysis seems to be an efficient method to point out medicinally important actinomycetes [13]. Mangrove ecosystems are very promising sources of actinobacteria [14]. These microorganisms have been identified as producers of antimicrobial compounds including cypemycin, neomycin, bottromycins, chloramphenicol and grisemycin [15]. In fact, our current research aimed to identify actinomycetes with antifungal properties. Referring to few previous reports [16,17], the present work focuses on identifying microbial leads for antifungal metabolite production and to screen the isolates for the presence of biosynthetic genes. The result reveals the occurrence of secondary metabolite genes in the majority of the isolates and enunciates their great probability in biosynthesizing novel metabolites, hence prioritizing the isolates for natural product discovery.

## 2. Results

### 2.1. Isolation and Identification of Mangrove Actinobacteria

**Figure 1:**
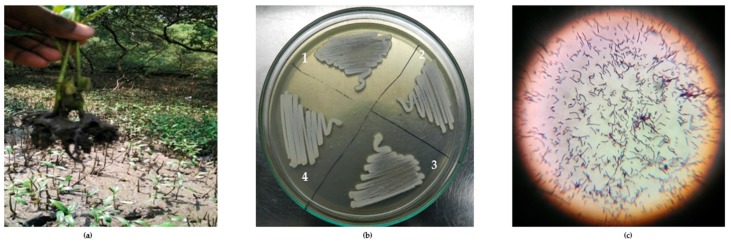
(**a**) Sampling of mangrove sediments; (**b**) Pure culture of Actinobacteria: l. VITGAP080, 2. VITGAP240, 3. VITGAP241, 4. VITGAP258; (**c**) Gram staining of *Streptomyces albidoflavus* (VITGAP241).

From 10 marine sediment samples collected, approximately 31 actinobacterial isolates were isolated and characterized. Figure 1 illustrates the isolation and morphology of the strains. Table 1 lists the strains obtained in this study along with the sampling location. From each of the sampling sites, sediments from different spots were collected to study the diversity and abundance of the organisms. In this study, 31 strains have been isolated from Muthupet (6 isolates) and from Andaman and the Nicobar Islands (25 isolates). Identification of the strains was done by 16S rDNA gene sequencing using the primers 27F and 1492R. Most of the actinobacteria isolated were found to fall under Streptomyces genus, while few of the strains were of the genera Rhodococcus (1), Corynebacterineae (1) and Actinomycetales (1). Significantly, the strains listed in Table 2 were identified as novel species based on the blast similarity of 16SrDNA gene sequences. In our study, among all media used, Starch casein agar media proved to be effective for the isolation of actinobacteria.

### 2.2. Genetic Screening

PCR screening of the actinobacterial strains revealed the presence of PKS type I, PKS type II and NRPS biosynthetic genes. Amplicons of desired size [PKS type I (bp-1.2kb), PKS type II (bp-500bp), NRPS (bp-700bp)] shows the presence of the biosynthetic gene. Table 3 lists the biosynthetic genetic potential of the actinobacterial strains. Among the 31 strains obtained, only 14 strains showed the presence of type I PKS gene and 6 of the strains showed the presence of type II PKS gene. Interestingly, all strains harboring type II PKS were also found to harbor type I PKS.

The biosynthetic potential of the actinobacterial strains reveals that type I PKS are more abundant than that of type II. Type I PKS was found to be harbored by about 20.8% of the actinobacterial strains, while type II PKS was found only with 8.3%. Remarkably, approximately 34.7% of strains showed the presence of NRPS gene. Almost all the strains harboring the genes belong to Streptomyces genus, while one of the strains revealing the presence of type I PKS is Rhodococcus. Interestingly, strains belonging to other genera, namely Corynebacterineae, did not harbor any of the studied genes.

### 2.3. Phylogenetic Analysis of the Actinobacteri Strains

**Figure 2:**
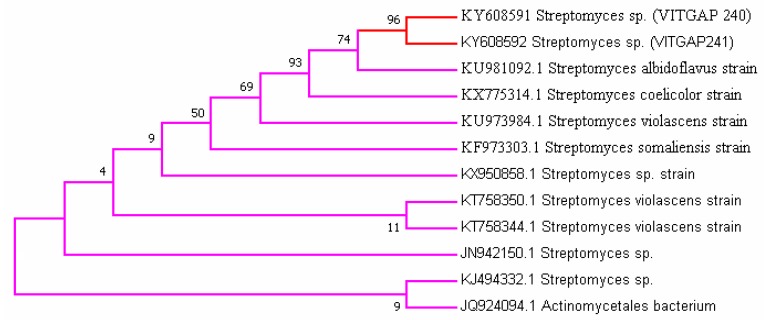
Phylogenetic tree based on 16S rRNA gene sequences of the strains.

**Figure 3:**
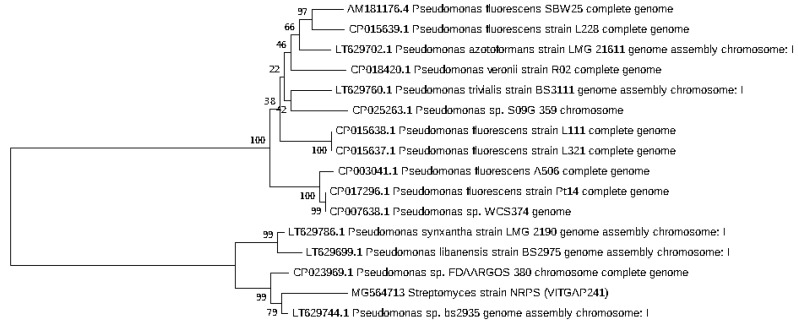
Dendrogram of NRPS (VITGAP241).

**Figure 4:**
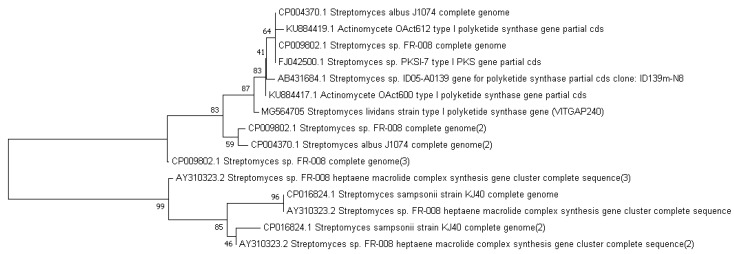
Dendrogram of Type I PKS (VITGAP240).

**Figure 5:**
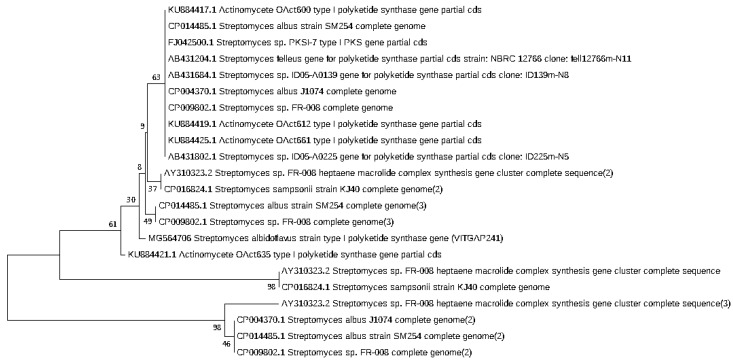
Dendrogram of Type I PKS (VITGAP241).

**Figure 6:**
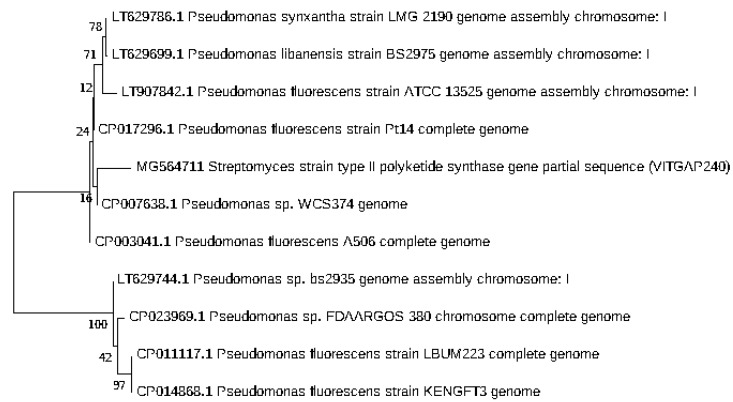
Dendrogram of Type II PKS (VITGAP240).

**Figure 7:**
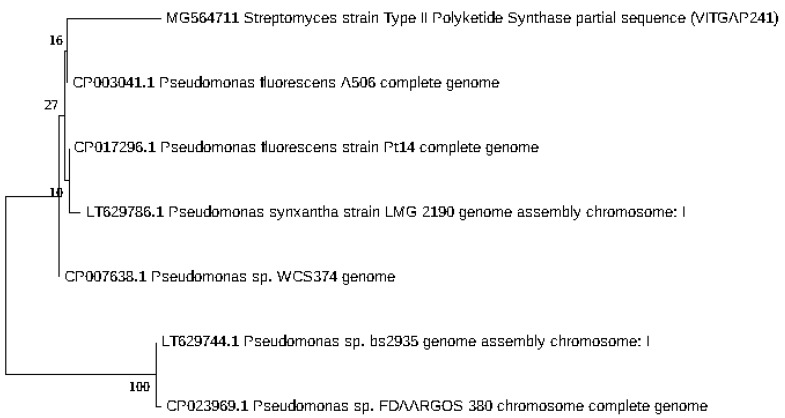
Dendrogram of Type II PKS (VITGAP241).

Phylogenetic analysis was performed by constructing the neighbor joining phylogenetic tree of all the strains to analyze the evolutionary relationship, and the distance was calculated by maximum-parsimony method using MEGA software (Figure 2). Based on the query coverage and the percentage of identity, the top aligned sequences were retrieved (Table 4). Significance of the branch order was determined by bootstrap analysis of 1000 replicates, see Figure 3, Figure 4, Figure 5, Figure 6 and Figure 7 (These figures depict the evolutionary relationships of taxa of the query sequences NRPS (VITGAP241), Type I PKS (VITGAP-240, VITGAP-241) Type II PKS (VITGAP-240, VITGAP-241). The optimal tree with the sum of branch lengths for each sequence are 1.31645770, 1.25726471, 1.52648453, 4.16276907, and 16.20030581 respectively. The percentage of replicate trees in which the associated taxa clustered together in the bootstrap test (1000 replicates) are shown next to the branches. The tree is drawn to scale, with branch lengths in the same units as those of the evolutionary distances used to infer the phylogenetic tree. The analysis involved 16, 16, 14, 11 and 7 nucleotide sequences. Codon positions included were 1st + 2nd + 3rd + Noncoding).

*Streptomyces* sp. (VITGAP240 & VITGAP241) share the conserved domain with a common ancestor. *Actinomycetales bacterium* is found be the new diverse species among the entire group of the *Streptomyces* family with minimum bootstrap value. *Streptomyces* sp. in two clades are common along with their closely related species, *Streptomyces violascens* strain.

### 2.4. Antifungal Activity Prospect Evaluations

**Figure 8:**
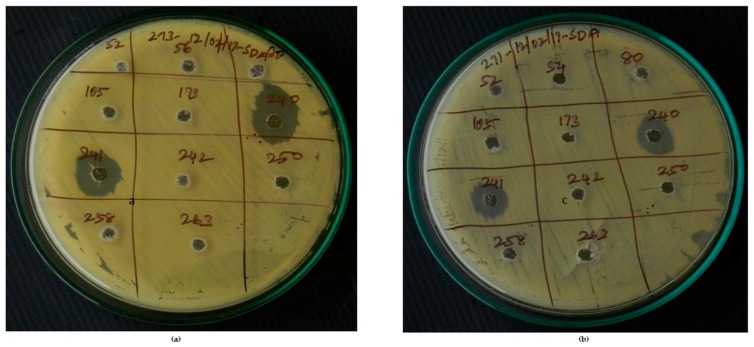
VITGAP240 & VITGAP241 showing significant activity against: (**a**) Clinical isolate 1 of *Candida albicans* (**b**) Clinical isolate 2 of *Candida albicans*.

Actinobacterial strains harboring biosynthetic genes were tested for their antifungal activity against 16 clinical isolates of *C. albicans* obtained from diagnostic centers in Tiruchirappalli, Tamil Nadu, India. Among the 14 bio-potential strains, only 2 of them (VITGAP240 and VITGAP241) exhibited an effective anti-candidal activity against the tested pathogens. Interestingly, these two bioactive strains possessed both type I and II PKS genes, hence attracting further attention. VITGAP241 also showed the presence of the NRPS gene. VITGAP241 exhibited more pronounced activity (18 mm diameter) than VITGAP240 (13 mm diameter). Figure 8 illustrates the bioactivity of the strains VITGAP240 and VITGAP241. 

Efficient antifungal activity was exhibited by the actinobacterial strains. As of our knowledge, this is the first report analyzing the antifungal potentiality of actinobacteria of Indian origin against *Candida albicans* (clinical pathogens). Though many of the strains possessing the genes were not functionally active, the two strains exhibiting bioactivity, harboured one of the biosynthetic genes supporting our hypothesis.

### 2.5. Antibiotic Sensitivity Assessment of Actinobacteria Strains

To assess the antibiotic sensitivity, the strains were screened against the following antibiotics: piperacillin, co-trimoxazole, ofloxacin, amikacin, erthyromicin, cefuroxime, tobramycin, ampicillin, tetracycline, ceftriazone, polmycin, nitrofurantin, rifampicin, clindamycin, netillin, ceftrazidime, novobiocin, aztreonam, cephotaxine, chloramphenicol, streptomycin, penicillin, methicillin, jancomycin, gentamycin, and vancomycin (Table 5). Based on PCR screening (Table 3), the potential strains which harbored PKS and NRPS gene and a few strains which did not show the presence of the genes were also chosen randomly for the assay to compare the strains. 

All the tested strains displayed significant sensitivity against most of the antibiotics used. The Highest sensitivity was obtained against netillin, novobiocin, cephotaxine (60% each), and lowest sensitivity was obtained against piperacillin, erthyromicin, nitrofurantin, chloramphenicol and jancomycin (6.6%). The strains exhibited resistance against the following antibiotics: aztreonam, streptomycin, penicillin, and methicillin. The strain VITGAP240 exhibited resistance to the following antibiotics: piperacillin, amikacin, erthyromicin, cefuroxime, ampicillin, polmycin, nitrofurantin, rifampicin, clindamycin, ceftrazidime, aztreonam, chloramphenicol, streptomycin, penicillin, methicillin, jancomycin and gentamycin. The strain VITGAP241 exhibited resistance to piperacillin, ofloxacin, erthyromicin, cefuroxime, tobramycin, ampicillin, ceftriazone, polmycin, nitrofurantin, clindamycin, ceftrazidime, aztreonam, streptomycin, penicillin and methicillin. Hence, the strains VITGAP240 and VITGAP241 proved to be promising and pharmacological important targets for the future, especially for antifungal based secondary metabolite profiling studies. 

### 2.6. GCMS Analysis and Screening of Bioactive Compounds

**Figure 9:**
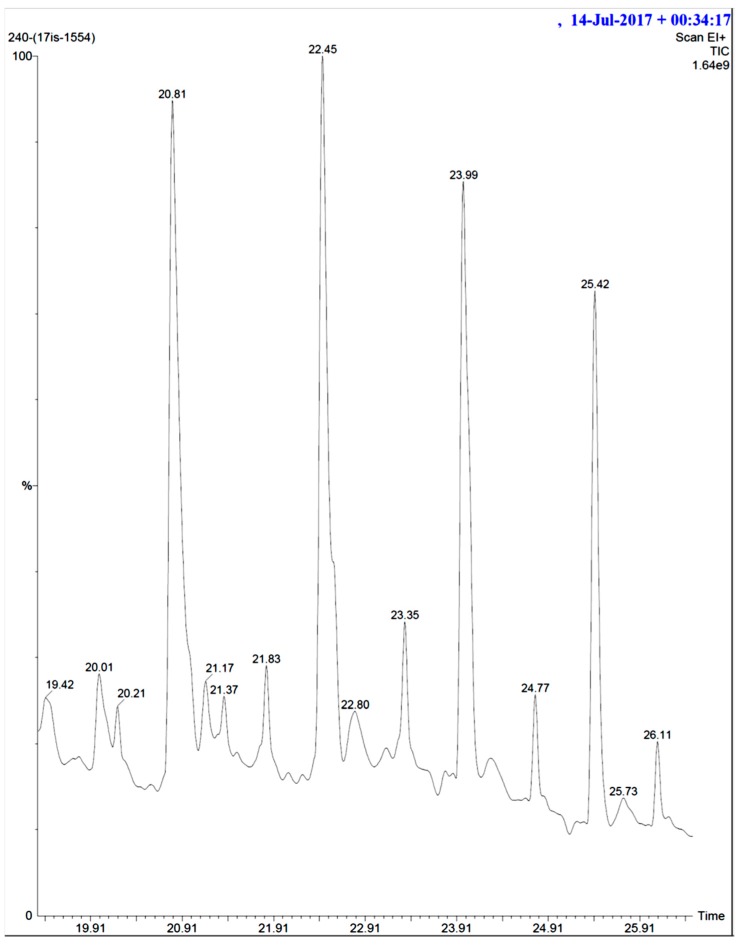
GCMS result of *Actinobacteria* secondary metabolite from marine mangrove sediment.

**Figure 10:**
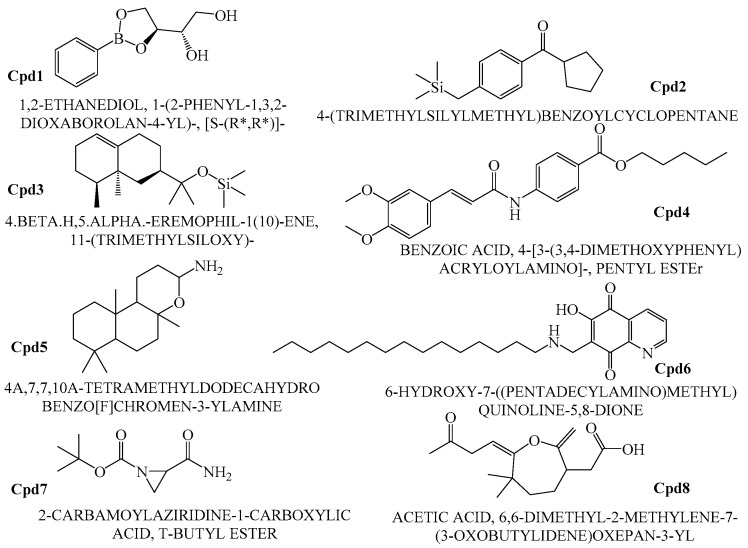
Heterocyclic compounds.

**Figure 11:**
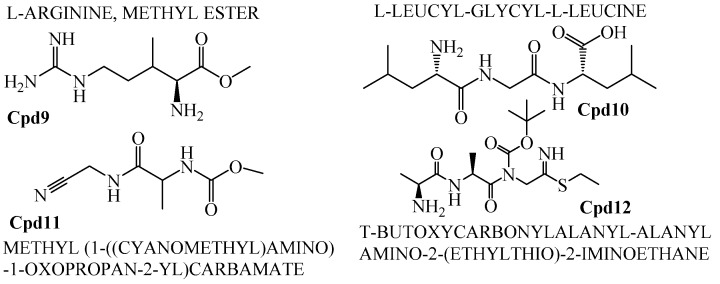
Peptide compounds.

**Figure 12:**
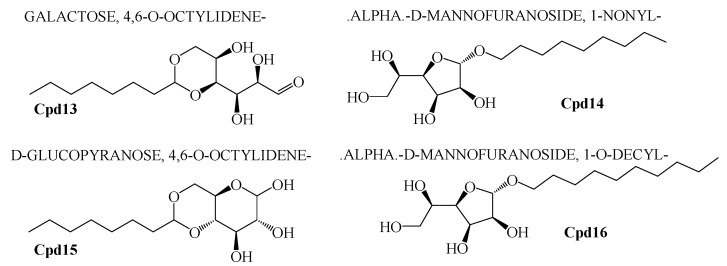
Polyketide compounds.

The crude extract of secondary metabolites was analyzed with the GCMS technique to identify the possible bioactive compounds’ availability. Figure 9 representatively illustrates a sample’s GCMS. Figure 10, Figure 11 and Figure 12 are the segregated compounds (Heterocyclic, Peptides, and Polyketides). Maximum of these compounds were found as unidentified or unpublished. Identified compounds were found with structural clarity and mostly equipped with functionally important scaffolds/groups. The confirmed nature allowed us to screen them as the most suitable medications against fungal infections.

### 2.7. Pharmacological Property Predictions of Screened Compounds

**Figure 13:**
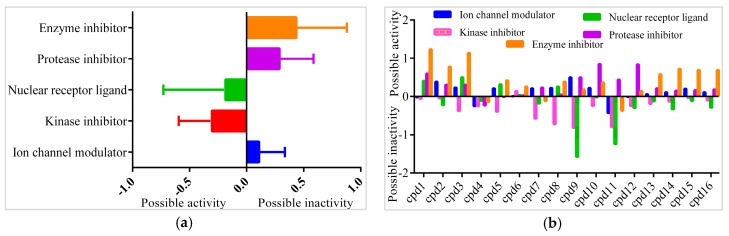
Bioactivity prediction results of isolated compounds from *Actinobacteria:* (**a**) Overall bioactivity of the compounds (**b**) Individual bioactivity of the compound.

In order to find the most suitable antifungal medications, the compounds that were categorized from GCMS studies were predicted for their possible biological activity potentials through Molinspiration and PASS (Figure 13). Among the segregated Heterocyclic (Cpd1–Cpd8) (Figure 10), peptides (Cpd9–Cpd12) (Figure 11), and polyketides (Cpd13–Cpd16) (Figure 12), a significant protease inhibition ability was found especially for peptide and polyketides. Particularly, the Molinspiration predictions revealed the strong Protease inhibitory potentials of all peptides (Cpd9–Cpd12) and polyketides (Cpd13–Cpd16) with a positive score range of 0.3 to 1. Apart from this, PASS prediction possible activity score of 0.922 (as NADPH peroxidase inhibitor) was found to have been established to the Heterocyclic compound; Cpd1 indicated its potential antifungal medicinal value. The opportunistic human fungal pathogen *Candida albicans* was already proven to use an NADPH oxidase enzyme (NOX) and reactive oxygen species (ROS) to regulate morphogenesis in an animal host [18,19]. So, this PASS score is a gateway to evaluate Cpd1 as a possible antifungal drug.

## 3. Discussion

Mangrove ecosystem remains untapped and thus seems to be a promising source as a rare actinobacteria enabling pipeline of novel natural products. Marine actinobacteria are well known to be valuable antifungal drug sources [20]. The present study has focused on targeting the biosynthetic genes in actinobacteria from the Andaman Islands, for production of novel antifungals. In this study, a potential strain has been identified from mangrove soil sediments which exhibits excellent inhibitory activity against clinical *C. albicans*. Biosynthetic genetic assessment was performed for the identified strain and then bioactive metabolites were extracted. To the best of our knowledge, this is the first study to evaluate the antifungal activity of Actinobacteria isolated from the Andaman Islands, against clinical pathogens of *C. albicans*. There are only a few reports about the actinobacteria from the Andaman islands, India which focuses on screening of enzymatic activity [21], antibacterial activity [22], and antifungal activity against multidrug resistant pathogens (*C. albicans*) [23]. Significantly, the study opens a new horizon by providing insights into the diversity and bio-potential of prominent strains isolated from mangrove ecosystem of the Andaman Islands.

Among all fungi, *C. albicans*, asymptomatically inhabits and affects various systems of healthy human beings and it is the most common microbe found in the oral and gastrointestinal tract in 40–60% cases of Candidiasis [24,25]. The occurrence of candidiasis even in HIV-infected and cancer patients, seems to clearly depict the increasing threat alarm [26]. Only rare species of Candida cause candidiasis due to the uncontrolled growth of fungus [27]. Henceforth, there is a thrust area in finding the prospective natural compounds against fungi.

Genes coding for secondary metabolites are highly conserved in the phylum of actinobacteria. The gene cluster analysis affords to identify the positive strains for the production of novel metabolites [28]. In this study, PCR screening for biosynthetic genes of the strains was performed. Degenerate PCR primers targeting conserved motifs in these genes helped to infer the biosynthetic potential of the strains [11]. The results revealed the existence of biosynthetic genes (PKS type I and II, NRPS), which helped in prioritizing the strains with inherent potential to synthesize secondary metabolites. NRPS positive strains are widely present, however, PKS type I and II are less frequent due to the lack of genes in those strains or less similarity of degenerate primers used in unusual domains present in the corresponding genes [29]. Among all the PKS and NRPS positive strains screened, only VITGAP240 and VITGAP241 were found to be potentially active against clinical isolates of *C. albicans*. Though gene-metabolite correlation helps in prioritizing the strains, the study indicates that all genetically positive strains were not functionally active because of cryptic-silent gene clusters [11]. Fermentation broths of two strains, namely VITGAP240 and VITGAP241, exhibited bioactivity against clinical isolates of *C. albicans*.

Among various compounds identified from the strain VITGAP241 crude extract through GC-MS analysis, only 16 compounds were selected based on the protease activity (generally acting as antifungal agents) [30] prediction using Molinspiration and PASS online tools. The obtained secondary metabolites of Actinomycetales highlights a potential source of numerous novel therapeutics with antifungal, antibacterial, antiviral, antiparasitic and antitumour etc. properties [28]. The compounds were grouped into polyketides, peptides and heterocyclic compounds. To the best of our knowledge the identified compounds from the strain *Streptomyces* sp. VITGAP241 have been reported for the first time by highlighting their extensive potential activity.

In addition to that, among the identified compounds, Cpd10 (l-Leucyl-Glycyl-l-Leucine) was found to have a carboxylic acid group which ensures the assured exchange of electrons to form hydrogen bonds within a minimal Å range. The anticipated interaction to achieve maximum expected therapeutic value, compound/drug dissolution, pharmacokinetic/pharmacodynamics and ADMET properties are made easy due to this facilitated functional group availability. Importantly, the Ligand-Receptor combinations requires -SH complex to inhibit the corresponding enzymes that are involved in the disease generation and development [31]. Hence, Cpd12 (T-Butoxycarbonylalanyl-Alanyl-Amino-2-(Ethylthio)-2-Aminoethane) was found to have established with a -S unit along with all other essential functional units which includes three carbonyl, methoxy, amino and methyl. Moreover, Cpd16 has the maximum protease inhibition activity of all other identified compounds. Further, polyketides that are widely substituted with -OH, -O, -CHO are normally involved in the bi-functional including electron donation and acceptation. This facilitates the internal temporary bridging between the compounds and the target proteins which depicts the promising medicinal value. The compounds Cpd13 (Galactose, 4,6-o-octylidene-), Cpd14 (.Alpha.-d-mannofuranoside, 1-nonyl-), Cpd15 (D-Glucopyranose, 4,6-o-octylidene-) and Cpd16 (.Alpha.-d-mannofuranoside, 1-o-decyl-) were commonly found to have -OH, -O, -CHO as their core functional units. The other group, Peptides, well-known protease inhibitors, were also established with -S, -NH, -O, -OH, -OMe and -Me. These are the best examples for mixed functional substituents i.e., electron donor, acceptor, functionally activators and deactivators that makes the compounds have a balanced interaction between the target proteins.

The strains were further allowed to assess the drug resistance which reveals the significant activity against the antibiotics. In particular, VITGAP240 exhibits the resistance against 17 antibiotics, whereas VITGAP241 exibits resistance to 14 antibiotics. This clearly indicates that these two bioactive strains encompass a wide spectrum of antimicrobials with their putative activity. The presence of multiple biosynthetic genes could be involved in the production of various compounds which shows the potential drug resistance [32]. Further investigation about the bioactive ingredients of potential strains shed light in understanding the relation between the biosynthetic gene and metabolites for the scientific community. Concomitantly, the strain Streptomyces sp. VITGAP241 harbor great potential to synthesize metabolites which could contribute to a large extent in drug discovery.

## 4. Materials and Methods 

### 4.1. Isolation of Marine Mangrove Actinobacteria

Marine mangrove sediments were collected aseptically in sterile plastic containers from Muthupet (3 October 2015) and Andaman, Nicobar Islands (20 February 2016), India. The collected samples were transferred to the lab, stored at 4 °C and processed within 2–3 days. One gram of collected wet sediment was suspended in 99 mL of 50% sterile sea water and kept for shaking at 120 rpm for 3 h in rotary shaker. The enriched sediment sample was subjected to serial logarithmic dilution in 0.8% saline down to 10^−4^. 0.1 mL of aliquot from each dilution; it was then plated in Starch casein agar, Actinomycetes isolation agar, International Streptomycetes project-2 and Bennett’s agar medium and incubated at 28 °C for 7–10 days [33]. Pre-treatment of the sediment samples was also done to avoid fungal growth, wherein, the sediment samples after a brief period of air drying, were subjected to heating at 65 °C for 20 min [34]. The pre-treated sediment samples were then processed in the same way as wet sediment processing. Actinomycetes were initially recognized by traditional morphological criteria including their characteristic leathery colonies, pigmentation including chalky white appearance, morphology of substrate and aerial hyphae [35].

### 4.2. Genomic DNA Extraction for Identification Through 16S rRNA Sequencing

The isolates obtained from the sediments were processed for the genomic DNA extraction, using HiPurA™ Streptomyces DNA Purification Kit (MB527, Himedia, Mumbai, India) for immediate use or for storage at −20 °C. Concentration and purity of the extracted DNA was then evaluated by running on Agarose gel and by NanoDrop (Thermo Scientific) readings. 

### 4.3. Amplification of 16S rRNA Gene and Sequencing

The genomic DNA obtained from the actinobacterial strains was further subjected to PCR amplification of 16S rRNA gene using the primers 27F-(5′-AGAGTTTGATCCTGGCTCAG-3′) and 1492R-(5′-ACGGCTACCTTGTTACGACTT-3′) [36]. The PCR mixture consisted of 5 µL of master mix (Ampliqon), 0.5 µL each of forward and reverse primer, 3.5 µL of ddH_2_O, 0.5 µL of template DNA (20 ng/µL) and the conditions were as follows: initial denaturation at 94 °C for 8 min; 30 cycles at 94 °C for 1 min, 57 °C for 1 min, and 72 °C for 2 min; and a final 8-min extension at 72 °C. Further detection of the PCR product was done by agarose gel electrophoresis. The purified PCR products were sequenced by MacroGen Co. (Seoul, Korea). Phylogeny prediction was done using MEGA 4.0. DNA sequences were deposited in NCBI with following accession numbers: KY608546-KY608550, KY608585-KY608609.

### 4.4. PCR Screening for Biosynthetic Genes

PCR amplifications with final volume of 100 µL were performed with following primers: PKS type I degenerate primers, K1F-(5′-TSAAGTCSAACATCGGBCA-3′) and M6R-(5′-CGCAGGTTSCSGTACCAGTA-3′) using the following conditions: initial denaturation of 95 °C for 10 min; 35 cycles of 95 °C for 30 s, 55 °C for 60 s, and 72 °C for 2 min; and a final extension at 72 °C for 10 min, PKS type II degenerate primers KSαF-(5′-TSGRCTACRTCAACGCSCACGG-3′) and KSβR-(5′-TACSAGTCSWTCGCCTGGTTC-3′) using the following conditions: initial denaturation of 95 °C for 10 min; 30 cycles of 95 °C for 30 s, 58 °C for 30 s, and 72 °C for 45 s; and a final extension at 72 °C for 10 min, for NRPS, A3F-(5′-GCSTACSYSATSTACACSTCSGG-3′) and A7R-(5′-SASGTCVCCSGTSCGGTAS-3′) using the following conditions: initial denaturation of 95 °C for 10 min; 35 cycles of 95 °C for 30 s, 61 °C for 45 s, and 72 °C for 1 min; and a final extension at 72 °C for 10 min [37,38]. Amplified fragments were then analyzed using 100 bp DNA ladder (HiMedia) in agarose gel (1.2%). The biosynthetic genes of the bioactive isolates were sequenced by MacroGen Co. (Seoul, Korea). The sequences were deposited in NCBI with following accession numbers: PKS type I—VITGAP240 (MG564705), VITGAP241 (MG564706), PKS type II—VITGAP240 (MG564711), VITGAP241 (MG564712), NRPS—VITGAP241 (MG564713).

### 4.5. Phylogenetic Analysis of the Strains

For the phylogenetic assessment, the gene sequences were aligned in Alignment Explorer of MEGA software, version 4 [39] using ClustalW preference. The trimming and verification of the sequence alignment were carried out by utilizing the MUSCLE (UPGMA) algorithm [40]. The neighbour-Joining [41] and maximum-parsimony methods were used to compute the evolutionary distances. Bootstrap analyses were performed with 1000 replications to evaluate the tree robustness [42]. 

### 4.6. Evaluation of Antifungal Activity

Actinobacterial strains harboring the biosynthetic genes were selected for evaluation of anti-candidal activity by agar well diffusion method [43]. 16 clinical isolates of *C. albicans* were obtained from diagnostic centers of Tiruchirappalli, Tamil Nadu. These isolates were inoculated in Sabouraud dextrose broth and incubated for growth until 10^6^ CFU/mL (0.5 McFarland) was obtained. 100 µL of the fungal suspensions were seeded onto Sabouraud dextrose agar plates and wells of 6 mm diameter were punched using sterile borer. The actinobacterial strains were inoculated in Starch casein broth and incubated at 28 °C, 150 rpm for 7 days. The culture supernatant was then collected after centrifugation of the culture at 10,000 rpm for 10 min. The culture supernatant was extracted with equal amount of ethyl acetate and subsequently evaporated to obtain the crude extracts. 50 µL of the actinobacterial culture extract were added to the wells and tested for anti-candidal activity. The plates were incubated at 28 °C for 24–48 h. The experiments were performed in triplicates. 

### 4.7. Antibiotic Susceptibility Test

Standard antibiotic discs (30 mcg/disc) were used to evaluate the antibiotic sensitivity blueprint of the isolates. The potential isolates were inoculated in starch casein broth and incubated at 28 ± 2 °C, 150 rpm for 7 days. The grown cultures were then plated on Starch casein agar plates and the antibiotic discs were placed on the top of the spread culture. The plates were then incubated at 28 °C for 24–48 h. The plates were then observed for the occurrence of inhibition zone and the diameters were measured [44]. Based on the zone formed, the isolates were considered either as susceptible (S), or resistant (R) for the antibiotics tested.

### 4.8. GCMS Results and Screening of Pharmacologically Important Bioactive Compounds 

In order to screen the bioactive or medicinally important secondary metabolite compounds, the Gas Chromatography Mass Spectrometry (GCMS) analysis was executed for the crude extract followed by the Thin Layer Chromatography (TLC) confirmations. TLC was performed using pre-coated silica-in alumina plate F_254_ (Merck) to separate the individual compounds from the non-volatile mixture (crude extract of the *Actinomycetes* sp.). Crude samples were spotted on the TLC plates and eluted from 0 to 100% *n*-Hexane/Ethyl acetate. The obtained spots were analyzed (TLC chamber) and noted for easy elution at suitable elution % using *n*-Hexane/Ethyl acetate (2:8) of individual spot via column chromatography. To determine the eluted individual compounds, GCMS analysis was carried-out using the Clarus 680 GC fused silica column, packed with Elite-5MS (5% biphenyl 95% dimethylpolysiloxane, 30 m × 0.25 mm ID × 250 μm df) and the components were separated using Helium as carrier gas at a constant flow of 1 mL/min. The injector temperature was set at 260 °C during the chromatographic run. The 1 μL of extract sample was injected into the instrument. The oven temperature was as follows: 60 °C (2 min); followed by 300 °C at the rate of 10 °C min^−1^; and 300 °C, where it was held for 6 min. The mass detector conditions were: transfer line temperature 240 °C; ion source temperature 240 °C; and ionization mode electron impact at 70 eV, a scan time 0.2 s and scan interval of 0.1 s. The spectrums of the components were compared with the database of spectrum of known components stored in the GCMS NIST (2008) library. ChemDraw (Version 15.0) was used to elucidate the structural confirmations of the bioactive compounds. The bioactive materials that were isolated from *Actinomycetes* sp. were categorized according the availability of functional groups under three major types i.e., polyketides, peptides and heterocyclic compounds. Their physical and chemical properties were unveiled. 

### 4.9. Pharmacological Property Predictions of Screened Compounds

The categorized compounds from GCMS analysis were predicted for their possible biological activity potentials through Molinspiration and PASS [45,46]. 

## 5. Conclusions

Marine actinobacteria are known to produce many secondary metabolites and its diversity in Indian marine ecosystem is being explored. Our present study evidently revealed the potential of actinobacteria isolated from Indian marine environments which allow the synthesis of a wide spectrum of antifungal compounds. A wide spectrum of the characterized bioactive compounds were the products of gene clusters that expressed coordinately. Moreover, the importance of PKS and NRPS genes for the expression of antifungal activity in marine actinobacteria has been established. Further, we found that the PCR based prescreening of target genes encoding for bioactive compound synthesis is one of the effective approaches for the detection of novel and active secondary metabolites. There are many questions with regard to the evolution and distribution of actinobacteria in the marine ecosystem, as well as unique sources for the isolation of novel actinobacteria which produce bioactive metabolites; this study depicts the potential of actinobacteria to synthesis bioactive compounds with the promising antifungal activity. Hence, further characterization of antifungal compounds generated by these actinobacteria could solve the emerging the problem of antibiotic resistance in *C. albicans*. Future studies should include isolation, identification and characterization of the bioactive compounds responsible for the bioactivity; insights about molecular interaction (between target receptor and potentially identified compounds); a suitable in vitro or in vivo model to validate medicinal effects of the compounds; establishment of Structure-Activity Relationship and finally ADMET, PK/PD and druggability validations.

## Figures and Tables

**Table 1:** Isolates obtained in this study and their accession ID.

Sequence-ID	Genbank Accession Numbers	Organism	Sampling Location	Latitude and Longitude
VITGAP080	KY608546	*Streptomyces rochei*	Muthupet, Thiruvarur, Tamil Nadu	10.39 N, 79.49 E
VITGAP095	KY608547	*Streptomyces variabilis*	Muthupet, Thiruvarur, Tamil Nadu	10.39 N, 79.49 E
VITGAP103	KY608548	*Streptomyces* sp.	Muthupet, Thiruvarur, Tamil Nadu	10.39 N, 79.49 E
VITGAP104	KY608549	*Streptomyces* sp.	Muthupet, Thiruvarur, Tamil Nadu	10.39 N, 79.49 E
VITGAP105	KY608550	*Streptomyces* sp.	Muthupet, Thiruvarur, Tamil Nadu	10.39 N, 79.49 E
VITGAP229	KY608585	*Streptomyces* sp.	Port Mout, Andaman and Nicobar Islands	11.40 N, 92.41 E
VITGAP231	KY608586	*Streptomyces* sp.	Port Mout, Andaman and Nicobar Islands	11.40 N, 92.41 E
VITGAP232	KY608587	*Streptomyces* sp.	Port Mout, Andaman and Nicobar Islands	11.40 N, 92.41 E
VITGAP233	KY608588	*Streptomyces clavuligerus*	Port Mout, Andaman and Nicobar Islands	11.40 N, 92.41 E
VITGAP235	KY608589	*Streptomyces* sp.	Port Mout, Andaman and Nicobar Islands	11.40 N, 92.41 E
VITGAP238	KY608590	*Streptomyces* sp.	Port Mout, Andaman and Nicobar Islands	11.40 N, 92.41 E
VITGAP240	KY608591	*Streptomyces lividans*	Corbyn, Andaman and Nicobar Islands	11.38 N, 92.44 E
VITGAP241	KY608592	*Streptomyces albidoflavus*	Corbyn, Andaman and Nicobar Islands	11.38 N, 92.44 E
VITGAP242	KY608593	*Streptomyces* sp.	Corbyn, Andaman and Nicobar Islands	11.38 N, 92.44 E
VITGAP244	KY608594	*Rhodococcus* sp.	Corbyn, Andaman and Nicobar Islands	11.38 N, 92.44 E
VITGAP245	KY608595	*Streptomyces violascens*	Corbyn, Andaman and Nicobar Islands	11.38 N, 92.44 E
VITGAP246	KY608596	*Corynebacterineae bacterium*	Corbyn, Andaman and Nicobar Islands	11.38 N, 92.44 E
VITGAP247	KY608597	*Streptomyces* sp.	Sippighat, Andaman and Nicobar Islands	11.36 N, 92.41 E
VITGAP248	KY608598	*Streptomyces* sp.	Sippighat, Andaman and Nicobar Islands	11.36 N, 92.41 E
VITGAP250	KY608599	*Streptomyces* sp.	Sippighat, Andaman and Nicobar Islands	11.36 N, 92.41 E
VITGAP253	KY608600	*Streptomyces* sp.	Wandoor Jetty, Andaman and Nicobar Islands	11.35 N, 92.37 E
VITGAP255	KY608601	*Actinomycetales bacterium*	Burmanalla, Andaman and Nicobar Islands	11.33 N, 92.43 E
VITGAP256	KY608602	*Streptomyces* sp.	Burmanalla, Andaman and Nicobar Islands	11.33 N, 92.43 E
VITGAP257	KY608603	*Streptomyces* sp.	Burmanalla, Andaman and Nicobar Islands	11.33 N, 92.43 E
VITGAP258	KY608604	*Streptomyces* sp.	Burmanalla, Andaman and Nicobar Islands	11.33 N, 92.43 E
VITGAP259	KY608605	*Actinomycetales bacterium*	Burmanalla, Andaman and Nicobar Islands	11.33 N, 92.43 E
VITGAP261	KY608606	*Streptomyces* sp.	Burmanalla, Andaman and Nicobar Islands	11.33 N, 92.43 E
VITGAP263	KY608607	*Streptomyces chumphonensis*	Burmanalla, Andaman and Nicobar Islands	11.33 N, 92.43 E
VITGAP270	KY608608	*Streptomyces* sp.	MundaPahad, Andaman and Nicobar Islands	11.29 N, 92.42 E
VITGAP271	KY608609	*Streptomyces* sp.	Kalapahad, Andaman and Nicobar Islands	11.36 N, 92.40 E

**Table 2:** Novel actinobacterial strains obtained in this study; 16S rDNAgene sequence similarities.

Isolate No.	Genbank Accession No. of the Isolates	Sampling Location of the Isolates	Closest Organism	Genebank No.	Similarity Percentage
VITGAP080	KY608546	Muthupet, Thiruvarur, Tamil Nadu	*Streptomyces rochei strain*	KP823705	96%
VITGAP095	KY608547	Muthupet, Thiruvarur, Tamil Nadu	*Streptomyces variabilis*	KU981101	95%
VITGAP 103	KY608548	Muthupet, Thiruvarur, Tamil Nadu	*Streptomyces* sp.	CP013142	95%
VITGAP 105	KY608550	Muthupet, Thiruvarur, Tamil Nadu	*Streptomyces* sp.	JQ009379	96%
VITGAP 235	KY608589	Port Mout, Andaman and Nicobar Islands	*Streptomyces* sp.	KX279534	83%
VITGAP 240	KY608591	Corbyn, Andaman and Nicobar Islands	*Streptomyces violascens*	KU973980	91%
VITGAP 253	KY608600	Wandoor Jetty, Andaman and Nicobar Islands	*Streptomyces* sp.	KU884356	94%
VITGAP 255	KY608601	Burmanalla, Andaman and Nicobar Islands	*Actinomycetales bacterium*	EU368818	88%
VITGAP 257	KY608603	Burmanalla, Andaman and Nicobar Islands	*Streptomyces* sp.	JF736620	97%
VITGAP 258	KY608604	Burmanalla, Andaman and Nicobar Islands	*Streptomyces* sp.	KR817750	87%
VITGAP 261	KY608606	Burmanalla, Andaman and Nicobar Islands	*Streptomyces* sp.	KX928494	92%
VITGAP 263	KY608607	Burmanalla, Andaman and Nicobar Islands	*Streptomyces chumphonensis*	NR_126175	94%
VITGAP 271	KY608609	Kalapahad, Andaman and Nicobar Islands	*Streptomyces* sp.	KT588654	92%

**Table 3:** Biosynthetic potential of the actinobacterial isolates.

Strains	PKS (Type I)	PKS (Type II)	NRPS
VITGAP080	+	+	+
VITGAP095			+
VITGAP105			+
VITGAP240	+	+	
VITGAP241	+	+	+
VITGAP242	+	+	+
VITGAP244	+		
VITGAP248			+
VITGAP250			+
VITGAP253			+
VITGAP255			+
VITGAP257			+
VITGAP258	+		+

‘+’ indicates the presence of biosynthetic gene.

**Table 4:** Query coverage and percentage of identity of the sequences.

Sequence	Query Coverage	% of Identity	No of Hits
NRPS_VITGAP241	99%	>83%	15
Type I PKS VITGAP-240	99%	>89%	15
Type I PKS VITGAP-241	80%	>79%	13
Type II PKS VITGAP-240	56%	>70%	10
Type II PKS VITGAP-241	68%	>67%	6

**Table 5:** Antibiogram profile of the strains (R-resistant, S-sensitive).

Antibiotics	Strains with the Biosynthetic Genes											Strains without the Biosynthetic Genes			
	80	95	240	241	242	105	248	250	253	255	258	103	105	261	263
Piperacillin	R	R	R	R	S	R	R	R	R	R	R	R	R	R	R
Co-Trimoxazole	R	S	S	S	S	S	S	S	R	R	S	R	R	R	R
Ofloxacin	S	S	S	R	S	R	S	R	R	S	R	R	S	S	R
Amikacin	S	S	R	S	S	R	S	R	R	R	R	R	R	S	R
Erthyromicin	R	S	R	R	R	R	R	R	R	R	R	R	R	R	R
Cefuroxime	S	S	R	R	R	R	S	R	R	R	R	R	R	R	R
Tobramycin	R	S	S	R	R	R	R	R	R	R	R	R	R	R	R
Ampicillin	R	S	R	R	R	S	S	R	R	R	S	R	R	R	R
Tetracycline	R	S	S	S	S	R	R	S	R	R	S	R	R	S	R
Ceftriazone	S	S	S	R	S	R	S	R	R	R	S	R	R	R	S
Polmycin	S	S	R	R	R	R	S	R	R	R	R	R	R	R	R
Nitrofurantin	R	R	R	S	R	R	R	R	R	R	R	R	R	R	R
Rifampicin	S	S	R	S	S	R	S	R	R	R	R	R	R	S	R
Clindamycin	R	R	R	R	S	R	R	R	R	R	R	R	R	R	R
Netillin	S	S	S	S	S	R	S	R	R	R	S	R	S	S	R
Ceftrazidime	R	S	R	R	S	R	R	R	R	R	R	R	R	R	R
Novobiocin	S	S	S	S	S	R	S	R	R	S	S	R	S	R	R
Aztreonam	R	R	R	R	R	R	R	R	R	R	R	R	R	R	R
Cephotaxine	S	S	S	S	S	R	S	S	R	R	S	R	R	S	R
Chloramphenicol	R	R	R	S	R	R	R	R	R	R	R	R	R	R	R
Streptomycin	R	R	R	R	R	R	R	R	R	R	R	R	R	R	R
Penicillin	R	R	R	R	R	R	R	R	R	R	R	R	R	R	R
Methicillin	R	R	R	R	R	R	R	R	R	R	R	R	R	R	R
Jancomycin	R	R	R	S	R	R	R	R	R	R	R	R	R	R	R
Gentamycin	S	S	R	S	R	R	S	R	R	R	S	R	S	S	R
Vancomycin	R	R	S	S	R	R	R	R	R	R	R	R	R	R	R
